# Implementation Strategies for Maternal Near-Miss Case Reviews in LICs and LMICs:  A scoping review protocol

**DOI:** 10.12688/wellcomeopenres.21398.1

**Published:** 2024-05-10

**Authors:** Vaibhav Agavane, Ramya Krishna B M, Pragati B Hebbar, Prashanth N S

**Affiliations:** 1 Institute of Public Health Bengaluru, Bengaluru, Karnataka, India

**Keywords:** Maternal health, Near-miss case review, Implementation strategies, Low-Income Countries, Middle-Income Countries, Scoping review.

## Abstract

**Background:**

Maternal mortality remains a persistent public health concern despite significant strides in reduction over the past few decades, with a global maternal mortality ratio (MMR) of 223 deaths per 100,000 live births in 2020, indicating a 34.3% decline over 20 years, with Low income countries (LICs) and Lower Middle-Income Countries (LMICs) bearing the major burden. Effective implementation of facility-based near-miss case reviews (NMCR), endorsed by the World Health Organization (WHO), faces challenges hindering progress, making exploring implementation strategies through a scoping review essential. This scoping review aims to identify and characterize implementation strategies employed in Low and Lower Middle- Income Countries to facilitate the implementation of facility-based NMCR.

**Methods:**

The scoping review will follow Arksey and O’Malley’s methodological framework, involving five stages: identifying the research question, selecting relevant studies, selecting data, charting, and summarizing the results. Electronic databases like PubMed, Embase, Web of Science, EBSCOhost - CINAHL Ultimate, and Ovid MEDLINE will be searched, supplemented by citation tracking. Rayyan will be used to screen and remove duplicates, with data charting conducted using Google Sheets. Two independent reviewers will conduct blinded screening, eligibility assessment, and inclusion phases. Reviewers will conduct Systematic data extraction independently using piloted forms, with discrepancies resolved through team discussion and consensus.

**Results:**

The review will identify and characterize implementation strategies employed to facilitate the implementation of facility-based near-miss case reviews in LICs and LMICs.

**Conclusions:**

The findings of this review will contribute to the understanding of implementing strategies for facility-based NMCR in LICs and LMICs. The review can help in designing interventions/programs to reduce maternal mortality and knowledge products.

## Introduction

### Background

Maternal mortality remains a persistent public health concern despite significant strides in reduction over the past few decades. Despite the development and advocacy of various evidence-based interventions, progress in reducing maternal mortality has been slow. In 2020, the global maternal mortality ratio (MMR) was estimated at 223 deaths per 100,000 live births, a reduction from 227 in 2015 and 339 in 2000, marking a 34.3% decline over 20 years ^1^. Low-income countries (LICs) and Low- and Middle-Income Countries (LMICs) experience the major burden of maternal mortality. In 2020, the MMR in LICs was estimated at 409 and LMICs at 232, whereas in High-Income Countries (HICs), it was 12 ^2^.

Amidst various strategies to enhance the quality of care in maternity services, The World Health Organization (WHO) and its partners have endorsed the Maternal Near-Miss Cases Review (NMCR) approach as part of the "Beyond the Numbers" strategy. A facility-based near-miss case review (NMCR) is an ongoing quality improvement process that enhances patient care and outcomes. It involves a review of the care provided to maternal near-miss cases, defined as “a woman who nearly died but survived a complication that occurred during pregnancy, childbirth or within 42 days (6 weeks) of termination of pregnancy” ^3^. Notably, when effectively implemented, facility-based NMCR has remarkably reduced maternal mortality ^4^.

However, effective implementation of facility-based near-miss case reviews in LMICs faces challenges, including lack of protocols, biased case selection, conflicting interests, inadequate training, resource limitations, blaming culture, hierarchical challenges, and unclear audit understanding ^5^. These barriers hinder the progress in reducing maternal mortality through NMCR.

Implementation science focuses on developing implementation strategies to overcome barriers to implementation and improve the effective implementation of evidence-based interventions. This approach involves understanding the context, identifying the barriers and facilitators to adoption, and designing implementation strategies specific to improve interventions' adoption and sustainability ^6^.

Implementation strategies utilized for effectively implementing NMCR in LICs and LMICs are not well described in the implementation research literature despite NMCR being identified as an effective intervention to reduce maternal mortality. This scoping review aims to identify and describe implementation strategies employed to facilitate the implementation of NMCR in LICs and LMICs.

### Rationale

As the efforts to reduce maternal mortality to achieve SDG goals in LICs and LMICs continue, NMCR can be an effective strategy for reducing maternal mortality in countries with high burden ^4^. While NMCR has been implemented in LICs and LMICs as an intervention to improve the quality of maternal care and reduce maternal mortality, a scoping review to identify and describe the strategies that have been implemented in such diverse contexts will be helpful to inform the future effective implementation of NMCR. This review can provide an opportunity to learn and adapt implementation strategies to diverse contexts to facilitate NMCR implementation and improve practice. 

### Ethics and dissemination

Ethics approval is not necessary for this scoping review as it involves the synthesis of existing literature. The intended dissemination of the findings plan includes publishing in a peer-reviewed journal and delivering presentations at relevant conferences to inform policymakers, researchers, students, and healthcare professionals about the implementation strategies for facility-based NMCR in LICs and LMICs.

## Protocol

### Objectives

The objective of this scoping review is to identify and characterize the implementation strategies utilized to enhance the implementation of facility-based NMCR in LICs and LMICs.

### Eligibility criteria

***Study characteristics.*** We will use the Population, Concept, and Context (PPC) framework to guide the development of our literature search strategy. Our review is interested in the Population (P) of pregnant women in LICs and LMICs, including those in the antenatal, intrapartum, and postpartum stages. This includes women who have experienced near-miss events during pregnancy, childbirth, or within 42 days of termination. For NMCR, the Concept (C) is implementation strategies. This includes specific methods or techniques to introduce, implement, and sustain NMCR within healthcare facilities.

We will include research articles from LICs and LMICs as per the latest World Bank classification of economies (Context). This will include healthcare facilities in urban and rural settings where we implement NMCR.

***Study design.*** Arksey and O’Malley’s methodological framework ^7^ will be followed to conduct the scoping review, and the steps are as follows:

Stage 1: Identifying the research question.
Stage 2: Identifying relevant studies.
Stage 3: Study selection
Stage 4: Charting the data
Stage 5: Collating, summarizing, and reporting the results

**Stage 1: Identifying the research question**

The scoping review question is, ‘What are the implementation strategies utilized to enhance the implementation of facility-based NMCR in LICs and LMICs?’. Curran *et al*. define implementation strategies as methods or techniques that enhance the adoption, implementation, and sustainability of a clinical program or practice ^8^. We will use this definition for our review.

**Stage 2: Identifying relevant studies**

Literature retrieval will occur from databases such as PubMed, Embase, Web of Science, EBSCOhost - CINAHL Ultimate, and Ovid MEDLINE. The review will include studies published after 2004, the year the WHO endorsed the NMCR approach as part of their ‘Beyond the Numbers’ strategy. Citation tracking will identify relevant articles.

**Stage 3: Study selection**

We will initially screen titles and abstracts of identified studies using Rayyan ^9^, a systematic review management software. We will remove duplicate records to ensure we assess each study only once. We will rigorously apply the inclusion and exclusion criteria during this phase to identify potentially relevant studies.

**Stage 4: Charting the data**

We will use a standardized data extraction form to systematically extract the required data from the included studies. This form will cover various aspects of the research articles (country of study, year of publication, outcomes studied, study design, etc.) and implementation strategies (name of the strategy, who delivered it, action target, etc.).

**Stage 5: Collating, summarizing, and reporting the results**

We will collate the synthesized data to provide a comprehensive overview of the implementation strategies. We will present them narratively, following the guidelines prescribed by the Preferred Reporting Items for Systematic Reviews and Meta-Analyses Extension for Scoping Reviews (PRISMA-ScR) ^10^. 

***Study setting.*** The review will include primary studies conducted in Low-income countries and Lower Middle-Income economies, as classified by the World Bank for the fiscal year 2024. 

***Inclusion and exclusion criteria.*** Our inclusion criteria will include peer-reviewed primary research studies encompassing experimental, quasi-experimental, or descriptive designs. We will focus on studies implementing facility-based NMCR, a criteria-based Audit conducted in LICs and LMICs. We will include studies published in English from 2004 onwards.

Our exclusion criteria will exclude grey literature such as conference abstracts, theses, dissertations, and reports. We will also exclude secondary reviews like systematic, literature, and narrative reviews.

### Key outcomes

This scoping review will focus solely on reporting the implementation strategies employed in NMCR as the primary outcome. The rationale for this decision lies in the review's specific aim to identify and describe the various implementation strategies utilized in implementing NMCR within healthcare facilities.

### Search strategy

The search strategy was developed by applying the PCC framework for scoping reviews. Following this, the search strategy was tailored to suit the specific requirements of each individual database included in the review (see Extended data for search strategy). This iterative process will involve adjusting search syntax, incorporating database-specific subject headings, and refining search strings to optimize retrieval of pertinent studies.

### Data management

Rayyan ^9^, a systematic review management software, will screen and remove duplicates. Additionally, Microsoft Excel will serve as the platform for data charting.

### Screening and data extraction

Two independent reviewers, RK and VMA, will carry out each phase of the review (screening, eligibility assessment, and inclusion) in a blinded manner. This approach will mitigate bias and will enhance the robustness of the study selection process. We will present the screening process using the PRISMA flow diagram ^10^. We will discuss and resolve any discrepancies in the screening process.

We will systematically carry out the data extraction process. Our reviewers will pilot data extraction forms to ensure consistency. Each reviewer will independently extract data. We will resolve any discrepancies through team discussion and consensus. We will extract the following data from each included source using our Data Extraction/Charting Form: Reviewer Name, Study Title, Aim of the study, Objectives of the study, Full-Text Link, Authors, Publication Year, Geographical study Location, Population/Participants, Study Setting, Number of Participants, Study Design, Implementation strategy/s utilized, Description of the Implementation Strategy, Components of the Implementation Strategy, Delivery of the Implementation Strategy, Implementation Context, Challenges Faced in Implementation, other Relevant Findings if any, Impact of Implementation Strategies on Outcomes, Funding, Name of the Funding Agency, Recommendations.

### Data synthesis and analysis

We will qualitatively synthesize and present the extracted data on implementation strategies. Our presentation of data will primarily use descriptive methods, including tables and figures, to describe the various implementation strategies we observe in the included studies. We will follow the Preferred Reporting Items for Systematic Reviews and Meta-Analyses (PRISMA) ^10^ guidelines extension for scoping reviews to summarize the identified implementation strategies. We will highlight key themes and patterns we observe across the included studies.

### Study status

This scoping review is currently in the third stage, which is the selection of study articles.

### Protocol and registration

The final protocol is registered with the Open Science Framework in March 2024; it is accessible with the DOI https://doi.org/10.17605/OSF.IO/HBXUY

## Conclusion

The review will contribute to understanding implementation strategies for facility-based NMCR in LICs and LMICs and can inform designing interventions/programs aiming to reduce maternal mortality, developing knowledge products for decision-makers, and identifying areas for further research.

## Data Availability

No underlying data is associated with this article. OSF: Implementation Strategies for Maternal Near-Miss Case Reviews in LICs and LMICs: A scoping review protocol https://doi.org/10.17605/OSF.IO/U4E9Y This project contains the following extended data: Search Strateg.pdf Data are available under the terms of the Creative Commons Zero "No rights reserved" data waiver (CC0 1.0 Public domain dedication).
