# Supplementary material for: Implementation Strategies for Maternal Near-Miss Case Reviews in LICs and LMICs:  A scoping review protocol
Source: Wellcome Open Res. 2024 May 10;9:247. [Version 1] doi: 10.12688/wellcomeopenres.21398.1 (PMC11310651; doi:10.12688/wellcomeopenres.21398.1)
Supplement: Peer review history

# Reviewer report: Reviewer response for version 1

**DOI:** 10.21956/wellcomeopenres.23666.r88215

**Published:** 2024-08-08

**Authors:** Almamy Malick Kante

**Affiliations:** 1 Bloomberg School of Public Health, Johns Hopkins University, Baltimore, Maryland, USA

This is an important and timely paper in the field of public health. However, the paper needs a review and edit from the authors. Since we did not have line numbers, I will just mention:

- Acronym such as NMCR and LICs and LMICs should be used when they appear first then in the rest of the paper.
- The last sentence of the Introduction/Background: the authors noted that "...implementing NMCR in LICs and LMICs are not well described ......... This scoping review aims to identify and describe implementation strategies employed to facilitate the implementation of NMCR in LICs and LMICs." It is important to review this sentence because if the authors said the implementation is not well described how are you going to describe it based on the literature review.
- Protocol / Eligibility criteria: replace PPC by PCC.
- Protocol / Stage 2: The Cochrane database can be an important resource be to included.
- Protocol / Stage 4: I would add in this list the WB classification in 2024 and the year of the study. Many countries have moved from LMIC to LIC during the period of the study. Also by using only the 2024 WB classification, there might be few countries that implemented this facility-based NMCR approach while they were ranked LIC/LMIC and they are no longer in this group. How are you going to deal with those countries.
- Protocol / Exclusion criteria: I believe there are few studies that have implemented community-based NMCR, you should mention that those studies will be excluded too.
- Conclusion: Should recommend how best to implement facility-based NMCR depending to where the country is LIC or LMIC. This means the analysis should probably be conducted by LIC versus LMIC.

Is the study design appropriate for the research question?

Yes

Is the rationale for, and objectives of, the study clearly described?

Yes

Are sufficient details of the methods provided to allow replication by others?

Yes

Are the datasets clearly presented in a useable and accessible format?

Not applicable

Reviewer Expertise:

Maternal and child health and mortality

I confirm that I have read this submission and believe that I have an appropriate level of expertise to confirm that it is of an acceptable scientific standard.

# Reviewer report: Reviewer response for version 1

**DOI:** 10.21956/wellcomeopenres.23666.r87033

**Published:** 2024-07-04

**Authors:** Brenda Sequeira Dmello

**Affiliations:** 1 Maternal and Newborn Health, Comprehensive Community Based Rehabilitation in Tanzania (CCBRT), Dar es Salaam, Dar es Salaam, Tanzania; 2 Department of Public Health, Global Health Section, University of Copenhagen, Copenhagen, Denmark

The authors are commended for clearly describing a protocol for conducting a scoping review to identify implementation strategies utilized by low-income countries (LICs) and lower-middle-income countries (LMICs) for Maternal Near Miss Case Review (NMCR).

 The topic is highly relevant. Based on a systematic review cited in this protocol (Lazzerini *et al.*, 2018), which did not have language or year restrictions and included a few additional databases, the number of articles retrieved was only nine.

 One reason for the limited number of articles may be that many LICs and LMICs are still grappling with maternal death audits. In many LMICs, implementing maternal death reviews also poses a challenge. The author team may need to consider this issue.

 Since the study aims to collate best practices for implementing NMCR, the author team could consider broader inclusion criteria.

 A few suggestions:

1. Widen the search criteria (year, language, databases).
2. Consider including grey literature (as NMCR are sometimes introduced as part of developmental interventions).
3. Include studies conducted in high-income countries (HICs). There could be valuable lessons for LMICs from geographies where NMCR is being practiced, with the proviso that context adaptation would be necessary.

If the authors intend to implement NMCR at a later stage, a Delphi review could provide valuable supplemental insights on effective implementation strategies.

Is the study design appropriate for the research question?

Yes

Is the rationale for, and objectives of, the study clearly described?

Yes

Are sufficient details of the methods provided to allow replication by others?

Yes

Are the datasets clearly presented in a useable and accessible format?

Not applicable

Reviewer Expertise:

Maternal and child health, urban health

I confirm that I have read this submission and believe that I have an appropriate level of expertise to confirm that it is of an acceptable scientific standard.
